# Blood eosinophil count and intensive care unit admission in patients hospitalized for acute exacerbation of COPD: a retrospective cohort study

**DOI:** 10.1186/s12890-026-04725-x

**Published:** 2026-09-11

**Authors:** Umran Ozden Sertcelik, Ahmet Sertcelik

**Affiliations:** 1 Department of Chest Diseases, Ankara Yıldırım Beyazıt University Faculty of Medicine, Ankara, Türkiye; 2 Department of Chest Diseases, Ankara Bilkent City Hospital, Üniversiteler Mah. 1604. Cad. No: 9, Ankara, 06800 Türkiye; 3 Department of Infectious Diseases and Clinical Microbiology, Division of Epidemiology, Ankara Yıldırım Beyazıt University Faculty of Medicine, Ankara, Türkiye; 4 Department of Infectious Diseases and Clinical Microbiology, Ankara Bilkent City Hospital, Ankara, Türkiye

**Keywords:** Pulmonary disease, Chronic obstructive, Eosinophils, Intensive care units, Disease exacerbation, Competing risks, Prognosis

## Abstract

**Background:**

Acute exacerbations of chronic obstructive pulmonary disease (COPD) are major clinical events associated with hospitalization, intensive care unit (ICU) admission, and increased mortality. Blood eosinophil count has emerged as a potential inflammatory biomarker in COPD; however, its role in predicting ICU requirement among hospitalized patients remains unclear.

**Methods:**

This retrospective cohort study included patients aged ≥ 40 years hospitalized with acute COPD exacerbation at Ankara Bilkent City Hospital between 2020 and 2024. COPD diagnosis was based on Global Initiative for Chronic Obstructive Lung Disease (GOLD) criteria. Blood eosinophil counts measured within the first 60 min of presentation to the emergency department were categorized as < 150 cells/µL, 150–299 cells/µL, and ≥ 300 cells/µL. ICU admission was defined as the primary outcome. Competing risk analysis using the Fine–Gray model was performed, with death as a competing event.

**Results:**

A total of 385 patients were included. Eosinophil groups were not significantly associated with ICU requirement. Clinical variables significantly associated with ICU admission included GOLD group, GOLD grade, modified Medical Research Council (mMRC) dyspnea score, long-term oxygen therapy (LTOT), nebulizer and non-invasive mechanical ventilation (NIMV) use, and need for NIMV at admission. In Fine–Gray models, GOLD group E was independently associated with increased ICU admission risk compared with GOLD A (subdistribution hazard ratio [sHR] = 3.35, 95% confidence interval [CI] = 1.57–7.15; *p* = 0.002), whereas eosinophil levels were not.

**Conclusion:**

In hospitalized patients with acute COPD exacerbations, blood eosinophil count — regardless of the threshold applied — was not independently associated with ICU admission after adjustment for disease severity and competing risks. GOLD group E emerged as the strongest independent predictor of ICU requirement in the same model. These findings suggest that clinical severity assessment, rather than eosinophil-based stratification, should guide risk assessment for ICU admission in this population.

**Clinical trial number:**

Not applicable.

**Supplementary Information:**

The online version contains supplementary material available at https://doi.org/10.1186/s12890-026-04725-x.

## Introduction

Acute exacerbations of chronic obstructive pulmonary disease (COPD) are major clinical events that significantly contribute to disease progression, healthcare utilization, and mortality worldwide. Hospitalization due to COPD exacerbations is frequently associated with poor outcomes, including the need for intensive care unit (ICU) admission, prolonged hospital stay, and increased short- and long-term mortality [1, 2]. Early identification of patients at high risk for severe exacerbations and ICU requirement remains a major clinical challenge and is essential for effective risk stratification and individualized treatment strategies.

In recent years, blood eosinophil count has emerged as a simple, inexpensive, and reproducible inflammatory biomarker in COPD, associated with exacerbation frequency, corticosteroid responsiveness, and clinical prognosis [3–5]. Blood eosinophil count has increasingly been used as a surrogate marker to guide inhaled corticosteroid (ICS) therapy in stable COPD. Current GOLD strategy documents recommend blood eosinophil thresholds (e.g., < 100 cells/µL, 100–299 cells/µL, and ≥ 300 cells/µL) to help identify patients who are more or less likely to benefit from ICS-containing regimens [2, 6, 7], and a meta-analysis of post-hoc RCT data showed that the exacerbation-reducing effect of ICS increases progressively with higher eosinophil thresholds, while low counts are associated with limited ICS benefit and increased pneumonia risk [8]. However, whether this same biomarker can be extrapolated to acute exacerbation settings—particularly for predicting exacerbation severity and the need for ICU-level care—remains far less established, highlighting an important knowledge gap that the present study aims to address.

Several studies have demonstrated that higher blood eosinophil levels are associated with a better response to corticosteroid therapy during acute exacerbations, whereas low eosinophil counts are more commonly linked to bacterial infection, increased systemic inflammation, and adverse outcomes [9, 10].

Despite these findings, the role of blood eosinophil count in predicting the severity of COPD exacerbations—particularly the need for ICU admission—remains unclear. While some studies suggest that eosinophilic exacerbations are associated with shorter hospital stays, lower treatment failure rates, and reduced mortality, other reports have shown inconsistent or conflicting results [11–13]. These discrepancies may be explained by differences in study design, eosinophil cut-off values, timing of measurement, prior corticosteroid exposure, and the presence of comorbidities. Moreover, data specifically addressing the association between eosinophil counts and ICU requirement in hospitalized COPD exacerbation patients are limited.

Clarifying whether eosinophil-based stratification can reliably predict ICU requirement in this population may have important clinical implications. Such an approach could facilitate early identification of high-risk patients, guide therapeutic decision-making, and improve allocation of critical care resources. Furthermore, identifying clinical and laboratory factors that influence the association between eosinophil count and ICU admission may enhance the applicability of eosinophil-guided risk assessment in routine clinical practice.

Therefore, the aim of the present study was to investigate the association between the blood eosinophil counts and ICU admission in patients hospitalized for acute COPD exacerbations.

## Materials and methods

### Study design

This study was designed as a retrospective cohort study. Consecutive patients who met the inclusion criteria during the study period were identified retrospectively through the hospital’s electronic medical record system, and their demographic, clinical, laboratory, and outcome data were extracted for analysis. Patients aged 40 years and older who were hospitalized with a diagnosis of acute exacerbation of COPD at Ankara Bilkent City Hospital between January 2020 and December 2024 were included. All eligible patients were followed from the time of admission until discharge, in-hospital death, or ICU admission, whichever occurred first, based on data documented in the medical records. For patients hospitalized more than once for AECOPD during the study period, only the first (index) qualifying admission was included in the analysis; subsequent admissions by the same patient during the study period were excluded to preserve the independence of observations. Ankara Bilkent City Hospital is a tertiary referral center that serves patients from Ankara as well as numerous national and international referrals. The hospital has a capacity of 958 intensive care unit beds and a total of 4,050 inpatient beds [14].

### Study population

The diagnosis of COPD was established according to the Global Initiative for Chronic Obstructive Lung Disease (GOLD) criteria. Patients admitted and followed in the hospital due to an acute exacerbation of COPD (AECOPD) were eligible for inclusion. AECOPD was defined when any of these International Classification of Diseases(ICD) − 10 (J44.1) code appeared. Exclusion criteria were as follows: (1) patients discharged without hospital admission (managed on an outpatient basis), (2) past or active malignancy, (3) hematological disorders, (4) parasitic infections, (5) patients without spirometry data, symptom assessment, or exacerbation history within the preceding year, (6) receipt of systemic corticosteroid therapy prior to hospital admission or (7) Patients who did not undergo complete blood count testing within the first 60 min of presentation to the emergency department for the index exacerbation.

### Data collection

Demographic characteristics (age and gender), smoking and biomass fuel smoke exposure, comorbidities, laboratory parameters, and clinical course data, as well as prior use of nebulizer therapy, long-term oxygen therapy (LTOT), and non-invasive mechanical ventilation (NIMV) before admission, were retrospectively obtained from the hospital’s electronic medical record system. Current and ex-smokers were combined into the category of “ever smoker,” while passive smokers and never smokers were combined into the category of “never smoker.

Blood eosinophil counts were determined from complete blood count analyses performed within the first 60 min of the patient’s presentation to the emergency department. This single, uniformly defined time point — emergency department presentation, rather than the time of subsequent ward admission — was used consistently throughout the study to eliminate ambiguity regarding the timing of eosinophil measurement. This early time window was selected a priori to minimize the confounding effect of corticosteroid administration on circulating eosinophil counts, since systemic corticosteroids can induce a rapid, dose-dependent reduction in blood eosinophils within hours of administration. Accordingly, blood samples were obtained as part of the routine admission laboratory work-up, prior to the initiation of any exacerbation-specific pharmacological treatment (including systemic corticosteroids, antibiotics, or escalation of bronchodilator therapy), in accordance with the standard admission protocol of the emergency department. Patients who had already received systemic corticosteroid therapy prior to admission, or whose complete blood count was not obtained within this 60-minute window, were excluded (see Exclusion Criteria), in order to ensure that eosinophil measurements reflected the patient’s baseline inflammatory status at presentation rather than a treatment-induced change. The need for NIMV during hospitalization was also recorded.

Patients were stratified according to blood eosinophil counts using two different classification approaches. First, patients were dichotomized into two groups based on an eosinophil threshold of 300 cells/µL (< 300 cells/µL and ≥ 300 cells/µL). Second, patients were further categorized into three groups according to eosinophil levels: <150 cells/µL, 150–299 cells/µL, and ≥ 300 cells/µL.

Modified Medical Research Council Dyspnea Scale (mMRC) scores [7, 15], COPD GOLD grades (according to severity of airflow obstruction, based on FEV1) [7], and COPD GOLD groups (A, B, or E, based on symptom burden and exacerbation history) [7] were recorded based on spirometry data, exacerbation history, and symptom assessment obtained from prior outpatient visits documented in the electronic medical record system. GOLD group assignment was based on the ABE assessment tool as defined in the 2025 GOLD.

### Outcome measures

The primary outcome of the study was the requirement for admission to the ICU during hospitalization. In the absence of a formal institutional protocol, ICU admission decisions were based on attending physician judgment, most commonly driven by acute respiratory failure with or without accompanying hemodynamic instability. Patients were classified into two groups based on clinical course: those who required ICU admission and those who did not. In-ward deaths were recorded as a competing risk. Time to event was defined as the interval from the date of hospital admission to either death or ICU admission, whichever occurred first. Patients who were discharged alive without requiring ICU admission were censored at the time of hospital discharge. No other censoring events, such as transfer to another hospital, were observed. To address potentially biased estimates arising from the censoring of competing events, the Fine-Gray subdistribution hazard model was applied.

### Statistical analysis

Statistical analyses were performed using Statistical Product and Service Solutions (SPSS) version 23.0 (IBM Corp., Armonk, NY, USA) and R statistical software version 4.5.2 (R Foundation for Statistical Computing, Vienna, Austria) with “*cmprsk*”, “*dplyr*”, “*ggplot2*” packages. Continuous variables were expressed as mean ± standard deviation or median with interquartile range, as appropriate, while categorical variables were presented as frequencies and percentages. For comparisons between the two eosinophil groups (≥ 300 cells/µL vs. <300 cells/µL) and between patients who required ICU admission and those who did not, the independent samples t-test was used when data followed a normal distribution, and the Mann-Whitney U test was applied otherwise. For comparisons across three peripheral eosinophil count groups (< 150 cells/µL, 150–299 cells/µL, and ≥ 300 cells/µL), one-way ANOVA was used when the normality assumption was met, and the Kruskal-Wallis test was employed when it was not. Post-hoc testing was not performed, as no statistically significant differences were identified. Comparisons of categorical variables between two groups were conducted using Pearson’s chi-square test or Fisher’s exact test, and comparisons across three groups were performed using the multiple-group chi-square test or the Freeman-Halton extension of Fisher’s exact test. Cumulative incidence graphs were plotted for intensive care unit admissions according to two- and three-category peripheral eosinophil count groups. In addition, the relationship between the groups was assessed using the Gray’s test.

Comparisons between groups were conducted using appropriate statistical tests according to data type and distribution. The association between eosinophil-based classifications and ICU admission was evaluated using competing risk analysis to account for competing events. The Fine and Gray subdistribution hazard model was applied, considering in-hospital mortality without ICU admission as a competing event. Subdistribution hazard ratios (sHRs) with 95% confidence intervals (CIs) were calculated. Age, gender, and disease severity were identified as potential confounders in the association between peripheral eosinophil count and ICU admission. Age and gender were included as confounders because they are associated factors with adverse clinical outcomes, including mortality, in patients with AECOPD [16].

Since disease severity-related variables such as GOLD group, GOLD grade, and mMRC score were correlated with one another, it was decided to include only GOLD group in the model in order to avoid the multicollinearity. In addition, sensitivity analyses were performed to assess the robustness of the primary models. In these analyses, GOLD group was replaced with mMRC score (Models 3 and 4) and GOLD grade (Models 5 and 6) as the measure of disease severity, and blood eosinophil count was evaluated using both three-category and two-category classifications in each of these models, respectively.

No data imputation was performed for missing values. A two-sided *p*-value < 0.05 was considered statistically significant.

### Ethical approval

The study protocol was approved by the Institutional Ethics Committee of Ankara Bilkent City Hospital(approval date: March 4, 2026, approval number: TABED 2-26-2024).Owing to the retrospective observationaldesign of the study and the use of anonymized patient data extractedfrom electronic medical records, the requirement for individualinformed consent was waived by the institutional review board.The study was conducted in accordancewith the principles of the Declaration of Helsinki.

## Results

**Fig. 1:**
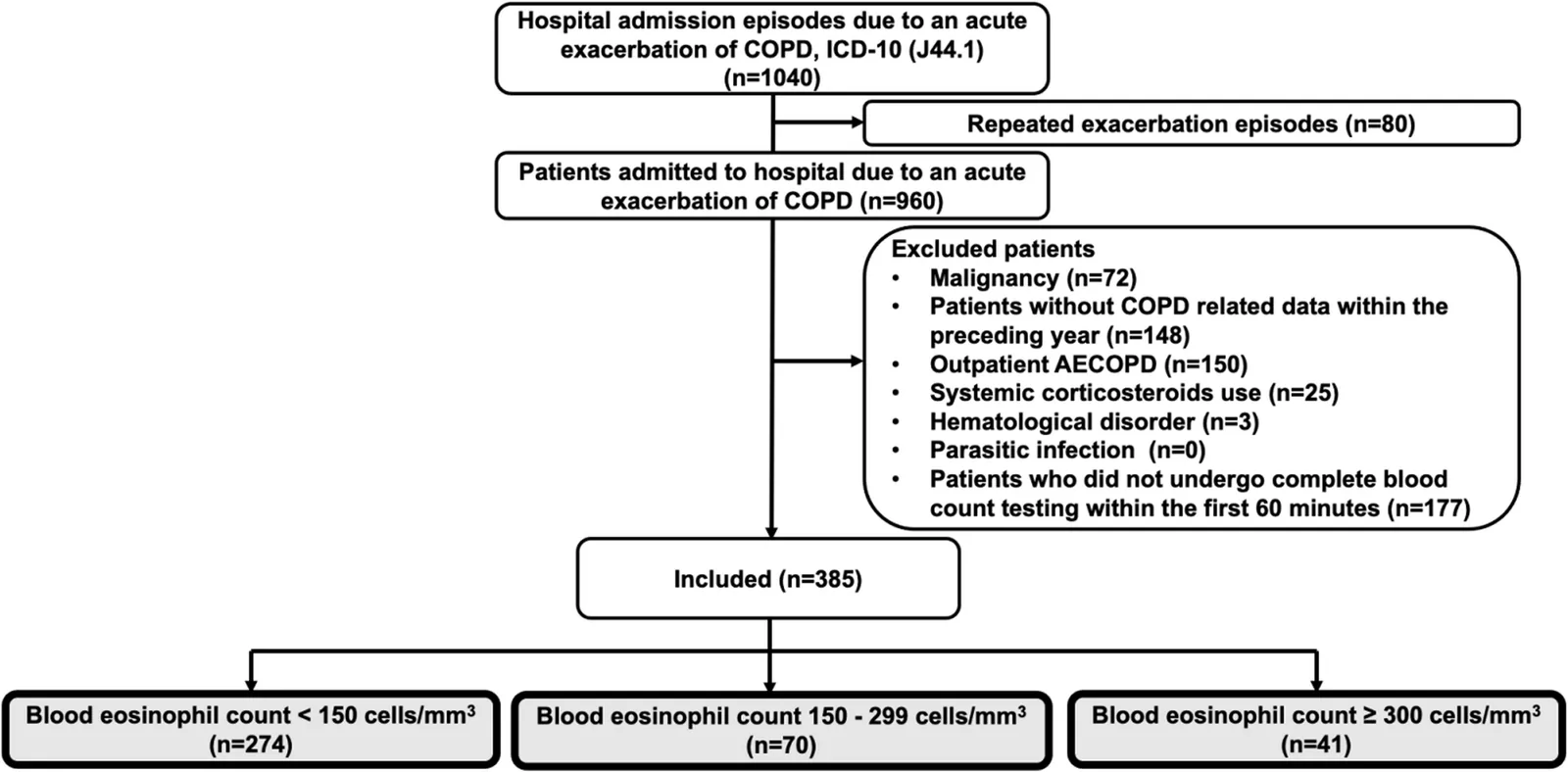
Flow chart of the study

Of 1,040 hospital admission episodes for acute exacerbation of COPD, 80 were excluded as repeated exacerbation episodes from the same patient, leaving 960 patients admitted to hospital due to AECOPD; after applying the predefined exclusion criteria, 385 patients were included in the final analytic cohort (Fig. 1). Patients were stratified according to blood eosinophil counts into three groups: <150/mm³ (*n* = 274), 150–299/mm³ (*n* = 70), and ≥ 300/mm³ (*n* = 41).The mean age of the study population was 70.2 ± 10.2 years, and 70.6% were male. There were no significant differences among eosinophil groups regarding age, gender distribution, duration of COPD, smoking characteristics, or time since smoking cessation, GOLD group, GOLD grade, mMRC dyspnea score, biomass fuel smoke exposure, comorbidities (Supplementary Table 1), and use of LTOT, NIMV, or nebulizer therapy and NIMV did not differ significantly between groups. Overall, 25.7% of patients were admitted to the ICU, while 74.3% were managed without ICU admission. ICU admission rates were numerically lower in patients with eosinophil counts ≥ 300/mm³, although this difference did not reach statistical significance (*p* = 0.336). Four patients (1.0% of the total cohort) were included as competing events because they died before the primary outcome, the ICU admission; this corresponded to 4 of 286 patients (1.4%) who were not admitted to the ICU. In contrast, in-hospital mortality among the 99 ICU-admitted patients was substantially higher, with 14 deaths (14.1%) (Table 1).

**Table 1:** Demographic, smoking characteristics, comorbidities, and clinical scores of patients hospitalized for acute exacerbation of COPD according to blood eosinophil counts

	All patients		Blood Eosinophil < 150/µL		Blood Eosinophil 150/ µL- 299/µL		Blood Eosinophil ≥ 300/µL		p1-value (eos < 300 vs. eos ≥ 300)	p2-value (eos < 150 vs. eos 150–299 vs. eos ≥ 300)
	*n*	Mean± SD Median (IQR)	*n*	Mean± SD Median (IQR)	*n*	Mean± SD Median (IQR)	*n*	Mean± SD Median (IQR)		
Age (year)	385	70.2 ± 10.2	274	69.9 ± 10.0	70	72.1 ± 10.9	41	68.5 ± 9.8	0.271	0.152
Years since diagnosis of COPD	385	9 (10)	274	10 (9)	70	8 (10)	41	9 (10)	0.980	0.984
Tobacco duration	313	40 (20)	222	40 (20)	56	40 (24)	35	40 (25)	0.587	0.781
Cigarettes per day	313	20 (0)	222	20 (0)	56	20 (0)	35	20 (20)	0.385	0.677
Years since quitting to smoke	165	10 (16)	115	10 (17)	30	9 (15)	20	9 (16)	0.950	0.954
	n (%)		n (%)		n (%)		n (%)			
Male gender	272 (70.6)		196 (71.5)		49 (70.0)		27 (65.9)		0.476	0.751
GOLD group									0.475	0.815
A	58 (15.1)		40 (14.6)		10 (14.3)		8 (19.5)			
B	133 (34.5)		98 (35.8)		24 (34.3)		11 (26.8)			
E	194 (50.4)		136 (49.6)		36 (51.4)		22 (53.7)			
GOLD grade									0.636	0.399
1	65 (16.9)		44 (16.1)		12 (17.1)		9 (22.0)			
2	104 (27.0)		79 (28.8)		17 (24.3)		8 (19.5)			
3	153 (39.7)		112 (40.9)		24 (34.3)		17 (41.5)			
4	63 (16.4)		39 (14.2)		17 (24.3)		7 (17.1)			
Smoking history									0.492	0.813
Current smoker	147 (38.2)		107 (39.1)		25 (35.7)		15 (36.6)			
Passive smoker	18 (4.7)		11 (4.0)		4 (5.7)		3 (7.3)			
Ex-smoker	167 (43.4)		116 (42.3)		31 (44.3)		20 (48.8)			
Never smoker	53 (13.8)		40 (14.6)		10 (14.3)		3 (7.3)			
Biomass fuel smoke exposure	260 (67.5)		181 (66.1)		50 (71.4)		29 (70.7)		0.726	0.623
Comorbidities	327 (84.9)		236 (86.1)		57 (81.4)		34 (82.9)		0.704	0.575
Long-term oxygen therapy	162 (42.1)		111 (40.5)		32 (45.7)		19 (46.3)		0.559	0.618
NIMV	61 (15.8)		44 (16.1)		11 (15.7)		6 (14.6)		0.822	0.973
Nebulizer	154 (40.0)		104 (38.0)		30 (42.9)		20 (48.8)		0.225	0.362
mMRC dyspnea score									0.593*	0.935*
0	16 (4.2)		12 (4.4)		3 (4.3)		1 (2.4)			
1	26 (6.8)		16 (5.8)		5 (7.1)		5 (12.2)			
2	82 (21.3)		59 (21.5)		16 (22.9)		7 (17.1)			
3	143 (37.1)		101 (36.9)		26 (37.1)		16 (39.0)			
4	118 (30.6)		86 (31.4)		20 (28.6)		12 (29.3)			
NIMV need during hospitalization	133 (34.5)		93 (33.9)		24 (34.3)		16 (39.0)		0.523	0.815
Outcome										
Admitted to ICU	99 (25.7)		75 (27.4)		16 (22.9)		8 (19.5)		0.336	0.468
Not admitted to ICU	286 (74.3)		199 (72.6)		54 (77.1)		33 (80.5)			
Discharged from the hospital	282 (73.2*)		197 (71.9)		53 (75.7)		32 (78.0)			
Died	4 (1.0*)		2 (0.7)		1 (1.4)		1 (2.4)			

*SD* Standard deviation, *IQR* Interquartile range, *COPD* Chronic obstructive pulmonary disease, *GOLD* Global initiative for lung diseases, *FEV1* Forced expiratory volume in the first second, *NIMV* Non-invasive mechanical ventilation, *ICU* Intensive care unit, *mMRC* modified Medical Research Council

*Percentages for the “Died” and “Discharged from the hospital” rows are calculated as a proportion of the total study population (*n* = 385)

There was no significant difference in age or gender distribution between patients admitted to the ICU and those not admitted (mean age = 71.2 ± 9.9 vs. 69.8 ± 10.3 years, *p* = 0.245; male gender: 69.7% vs. 71.0%, *p* = 0.809). However, the duration since COPD diagnosis was significantly longer in patients requiring ICU admission (median: 11.0 vs. 8.0 years, *p* < 0.001). Tobacco exposure duration, number of cigarettes per day, time since smoking cessation, and smoking status, were similar between groups (all *p* > 0.05). Likewise, biomass fuel smoke exposure and the presence of comorbidities did not differ significantly between ICU and non-ICU groups. These patients were more frequently classified as GOLD group E (72.7% vs. 42.7%, *p* < 0.001) and had more advanced GOLD grade, with a higher proportion of GOLD grade 3–4 (*p* < 0.001). Dyspnea severity assessed by mMRC score was significantly higher in patients admitted to the ICU, with more than half of these patients having an mMRC score of 4 (56.6% vs. 21.7%, *p* < 0.001). The need for LTOT was significantly more common in the ICU group (57.6% vs. 36.7%, *p* < 0.001), as was the use of NIMV (36.4% vs. 8.7%, *p* < 0.001) and nebulizer therapy (60.6% vs. 32.9%, *p* < 0.001). During hospitalization, the requirement for NIMV was markedly higher among patients admitted to the ICU compared with those not admitted (83.8% vs. 17.5%, *p* < 0.001) (Table 2).

**Table 2:** Demographic characteristics, smoking history, comorbidities, and clinical scores of patients hospitalized for acute exacerbation of COPD according to ICU admission status

	All patients		Admitted to ICU		Not Admitted to ICU		*p*-value
	*n*	Mean± SD Median (IQR)	*n*	Mean± SD Median (IQR)	*n*	Mean± SD Median (IQR)	
Age (year)	385	70.2 ± 10.2	99	71.2 ± 9.9	286	69.8 ± 10.3	0.245
Years since diagnosis of COPD	385	9 (10)	99	11.0 (10.0)	286	8.0 (9.0)	**< 0.001**
Tobacco duration	313	40 (20)	80	40.0 (20.0)	233	40.0 (20.0)	0.865
Cigarettes per day	313	20 (0)	80	20.0 (10.0)	233	20.0 (0.0)	0.101
Years since quitting to smoke	165	10 (16)	44	9.0 (14.0)	121	10.0 (17.0)	0.904
	*n* (%)		*n* (%)		*n* (%)		
Male gender	272 (70.6)		69 (69.7)		203 (71.0)		0.809
GOLD group							**< 0.001**
A	58 (15.1)		7 (7.1)		51 (17.8)		
B	133 (34.5)		20 (20.2)		113 (39.5)		
E	194 (50.4)		72 (72.7)		122 (42.7)		
GOLD grade							**< 0.001**
1	65 (16.9)		10 (10.1)		55 (19.2)		
2	104 (27.0)		8 (8.1)		96 (33.6)		
3	153 (39.7)		52 (52.5)		101 (35.3)		
4	63 (16.4)		29 (29.3)		34 (11.9)		
Smoking history							0.823
Ever smoker	314 (81.6)		80 (80.8)		234 (81.8)		
Never smoker	71 (18.4)		19 (19.2)		52 (18.2)		
Biomass fuel smoke exposure	260 (67.5)		72 (72.7)		188 (65.7)		0.200
Comorbidities	327 (84.9)		83 (83.8)		244 (85.3)		0.723
Long-term Oxygen Therapy	162 (42.1)		57 (57.6)		105 (36.7)		**< 0.001**
NIMV	61 (15.8)		36 (36.4)		25 (8.7)		**< 0.001**
Nebulizer	154 (40.0)		60 (60.6)		94 (32.9)		**< 0.001**
mMRC dyspnea score							**< 0.001**
0	16 (4.2)		1 (1.0)		15 (5.2)		
1	26 (6.8)		5 (5.1)		21 (7.3)		
2	82 (21.3)		9 (9.1)		73 (25.5)		
3	143 (37.1)		28 (28.3)		115 (40.2)		
4	118 (30.6)		56 (56.6)		62 (21.7)		
NIMV need during hospitalization	133 (34.5)		83 (83.8)		50 (17.5)		**< 0.001**

*SD* Standard deviation, *IQR* Interquartile range, *COPD* Chronic obstructive pulmonary disease, *GOLD* Global initiative for lung diseases, *FEV1* Forced expiratory volume in the first second, *NIMV* Non-invasive mechanical ventilation, *ICU* Intensive care unit, *mMRC* modified Medical Research Council

Bold *p*-values indicate statistical significance

**Fig. 2:**
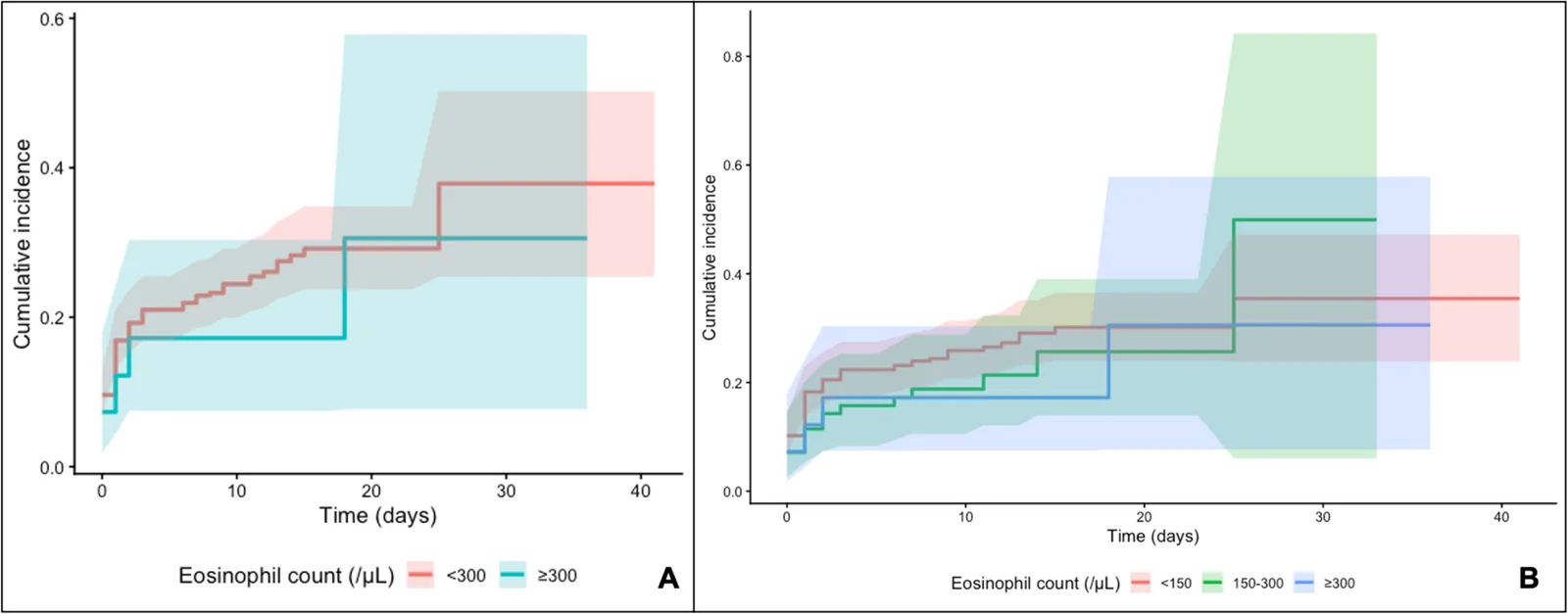
Cumulative Incidence Curves for ICU Admission Stratified by Blood Eosinophil Count [**A**: Two-group analyses (≥ 300 cells/µL vs. < 300cells/µL), **B**: Three-group analyses (< 150 cells/µL, 150–299 cells/µL and ≥ 300cells/µL)] Curves overlapped closely across eosinophil groups in both analyses, with non-significant Gray’s tests (**A**: *p* = 0.409; **B**: *p* = 0.513), indicating no association between eosinophil count and cumulative ICU admission risk over the follow-up period

Cumulative incidence curves overlapped closely across eosinophil groups throughout the follow-up period, both when eosinophil count was examined as two groups (Gray’s test *p* = 0.409) and when categorized into three groups (Gray’s test *p* = 0.513) (Fig. 2), indicating no association between eosinophil count and ICU admission risk. Based on the differences observed between patients admitted to the ICU and those not admitted, a multivariable competing risk analysis was conducted to identify independent factors associated with ICU admission. Considering that in-hospital death may preclude ICU admission, a Fine–Gray subdistribution hazard model was applied with death treated as a competing event. The model was adjusted for age, gender, GOLD group, and blood eosinophil count in order to evaluate their independent associations with ICU admission during hospitalization.

Given the 99 observed ICU admission events, in the multivariable Fine-Gray competing risk analysis adjusted for age, gender, GOLD group, and blood eosinophil count, GOLD group E was identified as an independent predictor of ICU admission. Compared with GOLD group A, patients classified as GOLD group E had a significantly higher risk of ICU admission in both models (Model 1: sHR = 3.35, 95% CI = 1.57–7.15, *p* = 0.002; Model 2: sHR = 3.35, 95% CI = 1.56–7.18, *p* = 0.002). Blood eosinophil count showed no evidence of a clinically important association with ICU admission, regardless of whether it was analyzed in three categories (< 150, 150–299, ≥ 300 cells/µL; Model 1) or dichotomized as ≥ 300 vs. <300 cells/µL (Model 2) (Table 3). To assess the robustness of these findings, sensitivity analyses were performed by replacing GOLD group with alternative measures of disease severity. When mMRC score was included in the model instead of GOLD group, mMRC dyspnea stage 4 was associated with ICU admission (Model 3: sHR = 9.422, 95% CI = 1.380–63.32, *p* = 0.022; Model 4: sHR = 9.435, 95% CI = 1.379–64.55, *p* = 0.022). Similarly, when GOLD grade was used in place of GOLD group, GOLD grade 4 was associated with ICU admission (Model 5: sHR = 3.435, 95% CI = 1.702–6.93, *p* = 0.001; Model 6: sHR = 3.364, 95% CI = 1.663–6.81, *p* = 0.001). Across all sensitivity models, blood eosinophil count continued to show no evidence of a clinically important association with ICU admission, regardless of whether it was analyzed in two or three categories (Supplementary Tables 2–3), consistent with the findings of the primary model.

**Table 3:** Fine-Gray competing risk regression analysis of factors associated with admission to intensive care unit

	Model 1			Model 2		
	sHR	95% CI	*p*-value	sHR	95% CI	*p*-value
Male gender	0.981	0.647–1.49	0.93	0.976	0.645–1.48	0.91
Age (years)	1.004	0.986–1.02	0.65	1.003	0.986–1.02	0.73
COPD GOLD Group						
B vs. A	1.266	0.543–2.95	0.59	1.269	0.543–2.97	0.58
E vs. A	3.345	1.565–7.15	**0.002**	3.350	1.563–7.18	**0.002**
Eosinophil count (/µL)						
150–299 vs. < 150	0.786	0.477–1.30	0.35			
≥ 300 vs. < 150	0.699	0.347–1.41	0.32			
≥ 300 vs. < 300				0.730	0.364–1.46	0.38

Model 1: A total of 385 patients were included in the analysis. Pseudo Log-likelihood = -550, pseudolikelihood ratio test = 26.2, df = 6

Model 2: A total of 385 patients were included in the analysis. Pseudo Log-likelihood = -550, pseudolikelihood ratio test = 25.4, df = 5

*sHR* Subdistribution hazard ratio, *CI* Confidence interval, *COPD* Chronic obstructive pulmonary disease, *GOLD* Global initiative for lung diseases

Bold *p*-values indicate statistical significance

## Discussion

This study examined the association between admission blood eosinophil counts and in-hospital ICU admission, in 385 patients hospitalized for acute exacerbation of COPD (AECOPD). Blood eosinophil count—whether categorized as < 150, 150–299, or > 300 cells/µL, or dichotomized at 300 cells/µL—showed no evidence of a clinically important independent association with ICU admission in a multivariable competing risk analysis. GOLD group E was identified as a strong and independent predictor of ICU admission, with more than a three-fold increased subdistribution hazard ratio compared with GOLD group A (sHR = 3.35, 95% CI = 1.57–7.15, *p* = 0.002). These findings remained consistent across sensitivity analyses in which GOLD group was replaced with mMRC dyspnea stage or GOLD grade as alternative measures of disease severity; blood eosinophil count continued to show no significant association with ICU admission in any of these models, regardless of whether it was analyzed in two or three categories (Supplementary Tables 2–3). This consistency across alternative model specifications strengthens confidence that, within the power available in this study, blood eosinophil count does not show a clinically important independent association with ICU admission in patients with AECOPD.

The role of blood eosinophils as a biomarker in COPD has gained substantial attention in recent years, particularly as a guide to inhaled corticosteroid therapy and for predicting exacerbation risk in stable disease. However, the prognostic value of admission eosinophil counts in hospitalized AECOPD patients remains controversial. Several studies have reported that lower eosinophil counts at admission are associated with worse in-hospital outcomes, including higher mortality rates and longer length of stay [17, 18]. A meta-analysis by Zhang et al. pooling data from 15 studies found that eosinophilic AECOPD, compared with non-eosinophilic AECOPD, was associated with a significantly lower risk of mortality (RR = 0.65, 95% CI = 0.54–0.77) and a shorter hospital stay, although readmission rates were modestly higher in the eosinophilic group [19]. In contrast, other studies, particularly those focusing on patients admitted to the ICU or with respiratory failure, have reported no significant association between eosinophil count and prognosis [20]. Our findings align with this latter body of evidence: across a broad, unselected inpatient population hospitalized for AECOPD, admission eosinophil count did not independently predict ICU admission.

A critical methodological contribution of this study is the application of the Fine–Gray subdistribution hazard model, which accounts for in-hospital death as a competing event. In studies of ICU admission during hospitalization, competing risks are a genuine concern because patients who die early in their hospitalization are inherently precluded from subsequent ICU admission [21]. Standard Cox regression, which censors competing events, can overestimate the cumulative incidence of ICU admission in such settings. By using the Fine–Gray approach, our analysis accounts for in-hospital death as a competing event when estimating the association between candidate predictors and ICU admission, thereby strengthening the validity of the conclusions. The consistent absence of a statistically significant association across both the two-group and three-group analyses supports the conclusion that blood eosinophil count was not independently associated with ICU admission in this cohort. As no formal analysis of incremental or discriminative value (e.g., AUC comparison, reclassification analysis) was performed, this study cannot determine whether eosinophil count adds discriminatory value beyond established severity measures.

The finding that GOLD group E was the sole independent predictor of ICU admission is clinically meaningful and consistent with the known prognostic importance of combined symptom burden and exacerbation history in COPD. GOLD group E, which captures patients with frequent or severe exacerbations, represents a high-risk phenotype defined by a history of recurrent acute deteriorations rather than spirometric severity alone. These patients are characterized by more advanced disease progression, greater functional impairment, and a higher burden of respiratory symptoms—features that plausibly increase the likelihood of clinical deterioration requiring intensive care. Prior studies have demonstrated that exacerbation history and symptom severity, as captured by the GOLD classification, are among the most robust predictors of adverse outcomes in AECOPD [2, 22]. Our data extend this observation to the specific outcome of in-hospital ICU admission using a methodologically rigorous competing risk framework.

Notably, the univariate disease-severity differences observed between the ICU and non-ICU groups (GOLD group E, GOLD grade, mMRC dyspnea score, LTOT, NIMV, and nebulizer use; see Results) were consistent with prior literature, and the markedly higher NIMV requirement in the ICU group further underscores the severity of respiratory compromise in this cohort. The longer duration of COPD diagnosis observed in the ICU admission group may additionally reflect cumulative disease burden and progressive loss of respiratory reserve over time [23]. Together, these findings support the conclusion that prior disease trajectory and functional status—rather than a single biomarker measured at admission—are the dominant determinants of ICU need.

The absence of a significant association between blood eosinophil count and ICU admission in our study may reflect several biological and clinical considerations. First, eosinophil counts obtained during AECOPD are subject to substantial suppression by systemic corticosteroids, infection, and the acute inflammatory milieu of exacerbation, potentially diminishing their signal-to-noise ratio as a biomarker at this clinical timepoint [24, 25]. Second, the heterogeneity of AECOPD triggers—including bacterial infections, viral infections, and non-infectious causes—may differentially affect eosinophil dynamics, making a universal threshold less informative in a mixed inpatient population. Third, ICU admission is predominantly driven by the need to manage acute respiratory failure and the requirement for escalation of ventilatory support, processes that may be more tightly linked to airway obstruction severity, acute physiological derangement, and pre-existing respiratory support requirements than to eosinophilic inflammatory phenotype.

The findings do not support an association between peripheral eosinophil count and ICU admission; rather, they suggest that classically disease severity–related scores are the stronger determinants of ICU-bound clinical trajectory. This study has several strengths, First, blood eosinophil count was measured within a standardized 60-minute window of emergency department presentation, and patients with documented pre-admission corticosteroid use were excluded a priori, minimizing treatment-related misclassification of eosinophil status. Second, we used a Fine-Gray competing risk model, appropriately accounting for in-hospital death as a competing event rahter than treating it as a censoring event, which avoids overestimating ICU admission risk. Finally, the analysis was based on a well-characterized single-center cohort with complete key variables for all included patients, and findings were supported by the sensitivity models using alternative eosinophil thresholds and severity measures.

These interpretations should be considered cautiously in light of the study’s limitations. Our study has several limitations. The retrospective, single-center design at a tertiary referral hospital precludes causal inference and may limit generalizability, and unmeasured confounders (e.g., arterial blood gas parameters, nutritional status, exacerbation trigger) together with selection bias from our exclusion criteria (pre-admission corticosteroid use, missing spirometry/symptom data) cannot be excluded. Eosinophil count was measured only once at admission; which does not capture known within-patient temporal variability [26] and undocumented or self-administered corticosteroid use before presentation cannot be fully excluded and may have further suppressed eosinophil counts in some patients. Furthermore, the relatively low overall in-hospital mortality rate (4.7%) in this cohort — driven mainly by the low mortality among patients not admitted to the ICU (1.4%), as in-hospital mortality was substantially higher among ICU-admitted patients (14.1%) — may reflect institutional management practices or case-mix effects that limit generalizability. Additionally, with only 99 ICU events observed, this study may be underpowered to detect small-magnitude associations between eosinophil count and ICU admission. Therefore, a clinically meaningful association cannot be entirely excluded on the basis of these findings, and the results should be interpreted as no evidence of a clinically important association rather than as definitive evidence against any relationship.

## Conclusion

This study demonstrates that blood eosinophil count at the time of AECOPD hospitalization was not independently associated with ICU admission after adjustment for disease severity and competing events. GOLD group E was recognized as an independent predictor of ICU admission, reinforcing the prognostic importance of exacerbation history and symptom burden in COPD management. These findings suggest that clinical escalation decisions in hospitalized AECOPD patients should continue to be guided by comprehensive disease-severity assessment, since eosinophil count was not independently associated with ICU admission in this cohort; whether it adds incremental discriminatory value beyond established severity measures was not formally tested and warrants further study.

## Supplementary Information

Supplementary Material 1.

## Data Availability

The datasets used and/or analyzed during the current study are available from the corresponding author on reasonable request.

## Abbreviations

AECOPD: Acute exacerbations of chronic obstructive pulmonary disease
CI: Confidence interval
COPD: Chronic obstructive pulmonary disease
FEV₁: Forced expiratory volume in one second
GOLD: Global Initiative for Chronic Obstructive Lung Disease
ICD: International Classification of Diseases
ICU: Intensive care unit
LTOT: Long-term oxygen therapy
mMRC: Modified Medical Research Council
NIMV: Non-invasive mechanical ventilation
sHR: Subdistribution hazard ratio
