# Supplementary material for: Blood eosinophil count and intensive care unit admission in patients hospitalized for acute exacerbation of COPD: a retrospective cohort study
Source: BMC Pulm Med. 2026 Sep 11;26:489. doi: 10.1186/s12890-026-04725-x (PMC13685114; doi:10.1186/s12890-026-04725-x)
Supplement: Supplementary file 1 [file 12890_2026_4725_MOESM1_ESM.docx]

Supplementary table 1. Comorbidity profile of the study population

n
%
Hypertension
239
62.1
Coronary heart disease
144
37.4
Diabetes mellitus
121
31.4
Congestive heart failure
97
25.2
Chronic renal failure
35
9.1
Atrial fibrillation
33
8.6
Hypothyroidism
25
6.5
Past cerebrovascular disease
20
5.2
Alzheimer’s Disease
8
2.1
Acute renal failure
2
0.5


Supplementary table 2. Sensitivity analysis: Fine-Gray competing risk regression analysis of factors associated with admission to intensive care unit, using mMRC score instead of GOLD group

Model 3
Model 4

sHR
95% CI
p-value
sHR
95% CI
p-value
Male gender
0.937
0.618 – 1.42
0.760
0.938
0.619 – 1.42
0.760
Age (years)
0.996
0.979 – 1.01
0.610
0.995
0.978 – 1.01
0.570
mMRC dyspnea score


1 vs 0
3.626
0.443 – 29.72
0.230
3.588
0.436 – 29.52
0.230

2 vs 0
1.783
0.238 – 13.38
0.570
1.773
0.564 – 0.236
0.580

3 vs 0
3.358
0.481 – 23.45
0.220
3.345
0.478 – 23.42
0.220

4 vs 0
9.422
1.380 – 63.32
0.022
9.435
1.379 – 64.55
0.022
Eosinophil count (/μL)


150 – 299 vs <150
0.847
0.521 – 1.38
0.500


≥ 300 vs <150
0.688
0.330 – 1.43
0.320


≥ 300 vs < 300


0.710
0.342 – 1.47
0.360
Model 1: A total of 385 patients were included in the analysis. Pseudo Log-likelihood = -542, pseudolikelihood ratio test = 41.9, df = 8
Model 2: A total of 385 patients were included in the analysis. Pseudo Log-likelihood = -542, pseudolikelihood ratio test = 41.5, df = 7
sHR: subdistribution hazard ratio, CI: confidence interval, mMRC: modified Medical Research Council


Supplementary table 3. Sensitivity analysis: Fine-Gray competing risk regression analysis of factors associated with admission to intensive care unit, using GOLD grade instead of GOLD group

Model 5
Model 6

sHR
95% CI
p-value
sHR
95% CI
p-value
Male gender
0.924
0.609 – 1.40
0.71
0.924
0.609 – 1.40
0.71
Age (years)
0.998
0.980 – 1.02
0.81
0.998
0.980 – 1.02
0.80
GOLD grade 


2 vs 1
0.474
0.191 – 1.17
0.11
0.478
0.192 – 1.19
0.11

3 vs 1
2.409
1.256 – 4.62
0.008
2.426
1.265 – 4.65
0.008

4 vs 1
3.435
1.702 – 6.93
0.001
3.364
1.663 – 6.81
0.001
Eosinophil count (/μL)


150 – 299 vs <150
0.740
0.450 – 1.22
0.24


≥ 300 vs <150
0.660
0.329 – 1.32
0.24


≥ 300 vs < 300


0.701
0.352 – 1.40
0.31
Model 1: A total of 385 patients were included in the analysis. Pseudo Log-likelihood = -542, pseudolikelihood ratio test = 42.0, df = 7
Model 2: A total of 385 patients were included in the analysis. Pseudo Log-likelihood = -542, pseudolikelihood ratio test = 40.7, df = 6
sHR: subdistribution hazard ratio, CI: confidence interval, GOLD: Global initiative for lung diseases
